# Complete genome sequence of an MDR and hypermucoviscous *Klebsiella pneumoniae* strain KP99 isolated from a 62-year-old patient in Dalian, China

**DOI:** 10.1128/mra.00432-26

**Published:** 2026-07-30

**Authors:** Fan Fan, Yuhua Hao, Tao Wang, Yuanting Gao, Pengni Chai, Shasha Luo, Mingke Song, Yihan Wu, Changxin Wu, Chonghua Hao, Zhiqiang Yang

**Affiliations:** 1 Key Laboratory of Chemical Biology and Molecular Engineering of Ministry of Education, Biomedicine and Health Laboratory in Shanxi Province, Institutes of Biomedical Sciences, School of Life Science, Shanxi University 12441, Taiyuan, China; 2 School of Health Care Technology, Dalian Neusoft University of Information 381860, Dalian, China; 3 Department of Infection, Affiliated Zhongshan Hospital of Dalian University 66562, Dalian, China; 4 Laboratory Medicine, The Affiliated People’s Hospital of Shanxi Medical University, Shanxi Provincial People’s Hospital, Taiyuan, China; Loyola University Chicago, Chicago, Illinois, USA

**Keywords:** *Klebsiella pneumoniae*, complete genome sequence, multidrug resistance (MDR), hypermucoviscous

## Abstract

We employed a PacBio-Illumina hybrid sequencing approach to sequence the strain KP99 isolated from the sputum of a clinical patient. Classified as ST592, KP99 carries multiple resistance genes (including *bla*_SHV-26_*, fosA5*, and *oqxA/B*) and harbors a hypervirulent factor system, including the *rmpA* gene linked to CPS and siderophore-associated genes.

## ANNOUNCEMENT

*Klebsiella pneumoniae*, a member of the *Klebsiella* genus in the *Enterobacteriaceae* family (1), is a common opportunistic pathogen. The growing prevalence of multidrug-resistant (MDR) (2) and hypervirulent *K. pneumoniae* strains poses a severe threat to global public health (3). In this study, we conducted complete genome sequencing on an MDR and hypermucoviscous (a phenotype associated with hypervirulence) (4) *K. pneumoniae* strain KP99 isolated from the sputum of a 62-year-old male patient hospitalized for acute exacerbation of chronic obstructive pulmonary disease (AECOPD) at a hospital in Dalian, China. After treatment with antibacterial agents to which KP99 was susceptible, the patient’s cough and dyspnea were alleviated, indicating that this pathogen plays a critical role in disease progression.

The sputum specimen positive for *K. pneumoniae* was primarily isolated on Columbia blood agar. Suspected colonies were purified on eosin methylene blue (EMB) agar, identified by VITEK MS (bioMérieux SA), and stored as glycerol stocks at −80°C. The strain exhibited a hypermucoviscous phenotype by a string test (string length >5 mm) (5). For genome sequencing, genomic DNA was extracted from a single colony cultured overnight in Luria-Bertani broth at 37°C using the TIANamp Bacteria DNA Kit (TIANGEN). For Illumina library preparation, genomic DNA was sheared into 400–500 bp fragments using a Covaris M220 Focused Acoustic Shearer, and libraries were constructed using the NEXTFLEX Rapid DNA-Seq Kit (Illumina). For PacBio library preparation, DNA was sheared into ~10 kb fragments, followed by end-repair and ligation of SMRT bell sequencing adapters (SMRTbell prep kit 3.0, Pacific Biosciences).

Whole-genome sequencing was performed on the Illumina NovaSeq 6000 platform, generating 4,543,064 raw paired-end reads (average depth 258.4×), and on the PacBio Sequel IIe platform, yielding 59,737 reads with an N50 of 9,667 bp (average depth 104.3×). The Illumina reads were quality-filtered with fastp v0.20.0 (6), and the cleaned short reads, together with PacBio HiFi reads, were assembled into a complete genome using Unicycler v0.4.8 (7), with the start point set to the *dnaA* gene. The assembly was subsequently polished with Pilon v1.22 (8) using short-read alignment. Genome annotation was performed using the National Center for Biotechnology Information (NCBI) Prokaryotic Genome Annotation Pipeline (PGAP) v6.10 (9). For subsequent genomic analyses, resistance gene screening, K-serotyping, and MLST were conducted with Kleborate v2.3.2 (10); plasmid replicon types were determined using KpVR v1.0 (11); and virulence genes were identified through the VFDB (12). A core-genome phylogenetic tree was constructed with Parsnp v2.0 (13), and 228 ST592-KL57 genomes were downloaded from NCBI. All tools were run with default parameters.

This *K. pneumoniae* strain was assigned to sequence type ST592, a rare MLST type (14), with a K locus type of KL57. Its assembled complete genome is 5,291,950 bp with 57.40% guanine-cytosine (GC) content comprising one chromosome and one plasmid. The resistance genes identified in this study are located on its chromosome, including *bla*_SHV-26_, *fosA5*, and *oqxA/B*. Additionally, KP99 harbors an almost complete virulence system in plasmid, including *rmpA*, siderophores-associated genes (*iutA*, *iroB/C/D/N*, *iucA/B/C/D*). Genome features for this strain are summarized in Table 1. The core-genome phylogenetic tree revealed that the clinical strain HK1020 (NCBI accession JAIBGS000000000) is the closest relative of strain KP99, differing by only 28 core-genome SNPs.

**TABLE 1:** Genome features of *K. pneumoniae* strain KP99

Feature	Value
Genome assembly	
Total length, bp	5,291,950
GC content (%)	57.40
Genome annotation	
Genes (total)	5,161
CDSs (total)	5,039
Genes (coding)	4,902
CDSs (hypothetical proteins)	267
Genes (RNA)	122
rRNAs	9, 8, 8 (5S, 16S, 23S)
tRNAs	86
ncRNAs	11
Chromosome	
Length, bp	5,073,754
GC content (%)	57.69
Drug resistance genes	*bla*_SHV-26_, *fosA5*, *OqxB*, *OqxA*
Virulence genes	*entF*, *acrB*, *icmF/tssM*, *fimD*, *clpV/tssH*, *fepA*, *iutA*, *tssF*, *vgrG/tssI*, *ompA*
Plasmid	
Length, bp	218,196
GC content (%)	50.47
Replicon types	IncHI3B(pNDM-MAR); IncFIB(K)_37
Virulence genes	*rmpA*, *iroB*, *iroC*, *iroD*, *iroN*, *iucA*, *iucB*, *iucC*, *iucD*, *iutA*, *TrbB*

The complete genome of ST592 strain KP99 provides a valuable genomic resource for clinically derived MDR and hypermucoviscous *K. pneumoniae*.

## Data Availability

The whole-genome sequence of strain KP99 has been deposited in GenBank under accession number JBWQXD000000000. Raw sequencing reads from PacBio Sequel platform are available in NCBI under accession number SRR38138837. Illumina raw sequencing reads are available under accession number SRR38081147, BioProject accession number PRJNA1444307, and BioSample accession number SAMN56744698. The strain HK1020 is available from NCBI under accession number JAIBGS000000000.
